# Allergy-Associated Acute Coronary Syndromes: Therapeutic Framework for Kounis Syndrome

**DOI:** 10.1016/j.jaccas.2026.107946

**Published:** 2026-04-16

**Authors:** Ruthra Ganeshanandha, Zhi H. Teoh, Rachael Hii, Derek P. Chew, Michael Leung

**Affiliations:** Victorian Heart Hospital, Monash Health, Clayton, Victoria, Australia

**Keywords:** acute coronary syndrome, allergy and immunology, interventional cardiology, Kounis syndrome

## Abstract

**Background:**

Kounis syndrome (KS) is a rare but clinically significant manifestation of acute coronary syndrome triggered by allergic, anaphylactic, or anaphylactoid reactions. Despite its potential severity, KS remains under-recognized in clinical practice.

**Objective:**

To present a case series illustrating the 3 subtypes of KS and to propose a management algorithm tailored to its unique pathophysiology.

**Methods:**

We describe 3 patients, each representing one of the KS subtypes: type 1 (coronary spasm without underlying disease), type 2 (plaque rupture in pre-existing coronary disease), and type 3 (stent thrombosis). All cases were managed in collaboration with cardiologists at the Victorian Heart Hospital, Monash Health.

**Conclusions:**

KS requires a distinct diagnostic and therapeutic approach compared to classic acute coronary syndrome. Our proposed algorithm aims to guide clinicians in recognizing and managing this complex syndrome effectively.

## Graphical Abstract

**Figure:**
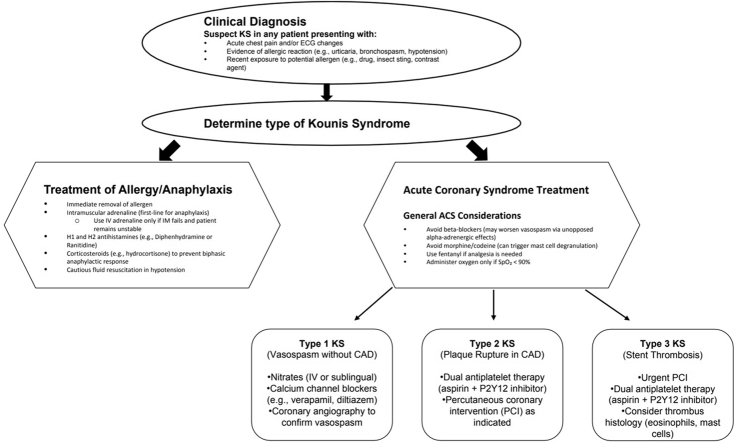

Kounis syndrome (KS) is a rare and often under-recognized complication of allergic reactions. Despite this, it has been reported that among patients admitted to the emergency department for allergic reactions, 3.4% were due to KS.^1^ Also known as “allergic angina syndrome,”^2^ KS is categorized into 3 types. Type 1 is characterized by coronary spasm with myocardial ischemia induced by an allergic event in patients with normal or nearly normal coronary arteries. Type 2 is characterized by plaque erosion or rupture and an acute myocardial infarction secondary to an acute hypersensitivity reaction. Type 3 is characterized by stent thrombosis from an acute allergic event.^3^^,^^4^

**Take-Home Messages**

• Kounis syndrome, defined as a concurrence of an acute coronary syndrome in the setting of an acute allergic event, is a rare and often missed complication of allergic reactions.
• There are 3 subtypes, as illustrated by the 3 cases in our case series; we have proposed a treatment algorithm, which differs significantly from classic acute coronary syndrome treatment.

In our case series, we present 3 patients illustrating each type of KS (Table 1). A timeline of the events of presentation for each case is provided in Table 2, Table 3, Table 4. We further discuss the management strategies of KS, which differ significantly from the treatment of classic atherosclerotic acute coronary syndrome (ACS), with a proposed therapeutic algorithm (Figure 1).

**Table 1:** Summary of Patients 1 to 3

Patient 1	
Presentation	52-year-old woman with anterior STEMI
Allergen	Aspirin
Management	Intracoronary GTN
Angiography	Pre-IC GTN Post-IC GTN
Diagnosis	Type 1 Kounis syndrome
Patient 2	
Presentation	67-year-old man with inferior STEMI
Allergen	Piperacillin-tazobactam (intraoperative)
Management	PCI to RCA (OCT confirmed plaque rupture)
Angiography	Pre-PCI to RCA Post-PCI to RCA
Diagnosis	Type 2 Kounis syndrome
Patient 3	
Presentation	59-year-old man with anterior MI (complicated by VT arrest)
Allergen	Bee stings (anaphylactic shock)
Management	IV adrenaline, DCCV, PCI to LAD (ISR)
Angiography	Pre-PCI Post-PCI
Diagnosis	Type 3 Kounis syndrome

DCCV = direct current cardioversion; IC = intracoronary; ISR = in-stent restenosis; IV = intravenous; GTN = glyceryl trinitrate; LAD = left anterior descending artery; MI = myocardial infarction; PCI = percutaneous coronary intervention; RCA = right coronary artery; STEMI = ST-segment elevation myocardial infarction; VT = ventricular tachycardia.

**Table 2:** Case 1: Timeline of Events

Time	Events
Day 1 admission	A 52-year-old woman presented with typical chest pain worsening over 2 wk. ECG showing lateral lead T-wave inversion with troponin rise from 34 to 77 ng/L. Had interval worsening of chest pain and asthma symptoms associated with inadvertent aspirin administration by paramedics (known allergy). Emergent LHC: Coronary spasm of LAD/LCx, improved with intracoronary GTN. Admitted to CCU for monitoring. Ticagrelor commenced.
Day 2	Ongoing mild left chest pain. Commenced on amlodipine and GTN infusion.
Day 3	Chest pain and shortness of breath resolved. GTN infusion ceased, switched to GTN patch. Amlodipine changed to diltiazem. TTE: Normal LVEF with mild distal LAD territory hypokinesis. No valvular dysfunction.
Day 4	Further chest pain (atypical; different to presentation) noted radiating to neck. Cardiac MRI scheduled. GTN patch ceased. Had further typical chest pain overnight radiating to left shoulder, with ECG showing ST-segment depression on anterolateral leads. Restarted on GTN infusion.
Day 5	Chest pain improved, commenced on nicorandil 10 mg twice daily. GTN infusion continued. Remained inpatient awaiting cardiac MRI to assess for myocarditis.
Day 6	Free of chest pain. GTN infusion weaned off. Noted to have fever and productive cough overnight with new oxygen required. Chest x-ray noted left basal collapse/consolidation and pleural effusion. Commenced on augmentin to cover for hospital-acquired pneumonia. Given emergent intravenous Lasix.
Day 7	Pain free. History revisited, noted to have penicillin and aspirin allergies. Had been unwell for around 1 mo. Given augmentin in the community for treatment of otitis media, which precipitated initial chest pain episodes. Further aspirin load by paramedics during presentation triggered worsening chest discomfort. Rheumatology team referred. Augmentin switched to cefuroxime. Cardiac MRI with LGE: subendocardial and midmyocardial delayed enhancement of the basal and midinferior segments.
Days 8-9	Rheumatology review. Impression of EGPA initially. Differential of eosinophilia associated coronary vasospasm (Kounis). Vasculitis screen sent off. Remained clinically stable.
Day 10	Vasculitic screen returned negative. Diagnosis of allergy-associated coronary vasospasm (Kounis syndrome) established. Discharged home well with immunology follow-up.

CCU = cardiac care unit; ECG = electrocardiogram; EGPA = eosinophilic granulomatosis with polyangiitis; GTN = glyceryl trinitrate; LAD = left anterior descending artery; LCx = left circumflex artery; LGE = late gadolinium enhancement; LHC = left heart catheterization; LVEF = left ventricular ejection fraction; MRI = magnetic resonance imaging; TTE = transthoracic echocardiography.

**Table 3:** Case 2: Timeline of Events

Time	Events
Day 1	A 66-year-old man, elective admission for laparotomy and adhesiolysis with intraoperative ablation of segment 4a HCC. Intravenous Tazocin administered given iatrogenic diaphragmatic breach and potential contamination. Intraoperative hypotension noted. Commenced on adrenaline infusion and procedure completed. Admitted to ICU postprocedure. Intubated and sedated. Tryptase levels sent (83.9 μ/L) Noted ST-segment elevation on inferior leads with POCUS showing hypokinesis of posterior wall. Urgent cardiology review: Transferred to Victorian Heart Hospital for emergent LHC. RCA lesion with plaque rupture confirmed on OCT imaging. Successful PCI to RCA.
Days 2-6	Lactate downtrending. Intravenous meropenem ongoing. (clindamycin ceased). Noted new AF. Transferred back to ICU at Monash Clayton for hepatobiliary team surgical reviews. Ongoing monitoring of neurological state, planning extubation. Adrenaline infusion gradually weaned. NG feeds commenced. Stable from cardiac perspective.
Days 7-14	Successful extubation on day 7, adrenaline infusion ceased. Transferred to ward. Remained under care of hepatobiliary team with daily cardiology consults. Referred to immunology team for ongoing outpatient reviews. Ongoing allied health reviews for reconditioning. Cleared for discharge on day 15. Tryptase trend: 83.9 μ/L (intraoperative) > 68.9 μ/L (2 h after initial event) > 13.2 μ/L (day 2) > 10.5 μ/L (day 8) (normal range: 0-11.4 μ/L)
Day 15	Discharged home well.

AF = atrial fibrillation; HCC = hepatocellular carcinoma; ICU = intensive care unit; LHC = left heart catheterization; NG = nasogastric; OCT = optical coherence tomography; PCI = percutaneous coronary intervention; POCUS = point-of-care ultrasound; RCA = right coronary artery.

**Table 4:** Case 3: Timeline of Events

Time	Events
Day 1	A 59-year-old man, brought in by ambulance for anaphylaxis (12 bee stings) followed by unresponsive episode, stridor, unrecordable blood pressure. Responded to repeated doses of IM and IV adrenaline. VF noted afterward and received shock ×3. Upon ROSC, ECG showed anterolateral ST-segment elevations with corresponding chest pain on arrival to emergency department. STEMI code – LHC: Severe proximal LAD stent 80% stenosis. PCI to ostial LAD with 2 overlapping DCBs and POBA, with no significant residual narrowing. OCT assessments before and after PCI showed good result. Tryptase levels: 128 μ/L (on arrival) > 48 μ/L (4 h) > 20.4 μ/L (10 h) > 9.0 μ/L (15 h) (range, 0-11.4 μ/L).
Days 2-4	Clinically stable. No supports required. Free of chest pain. TTE: Concentric LV hypertrophy with normal ejection fraction despite apical infarct/aneurysm. Mild MR. Mild LA enlargement. No LV thrombus.
Day 5	Discharged home well. Follow-up arranged with cardiology and immunology teams.

DCB = drug-coated balloon; ECG = electrocardiogram; IM = intramuscular; IV = intravenous; LA = left atrial; LHC = left heart catheterization; LV = left ventricular; MR = mitral regurgitation; OCT = optical coherence tomography; PCI = percutaneous coronary intervention; POBA = plain old balloon angioplasty; ROSC = return of spontaneous circulation; TTE = transthoracic echocardiography; VF = ventricular fibrillation.

**Figure 1:**
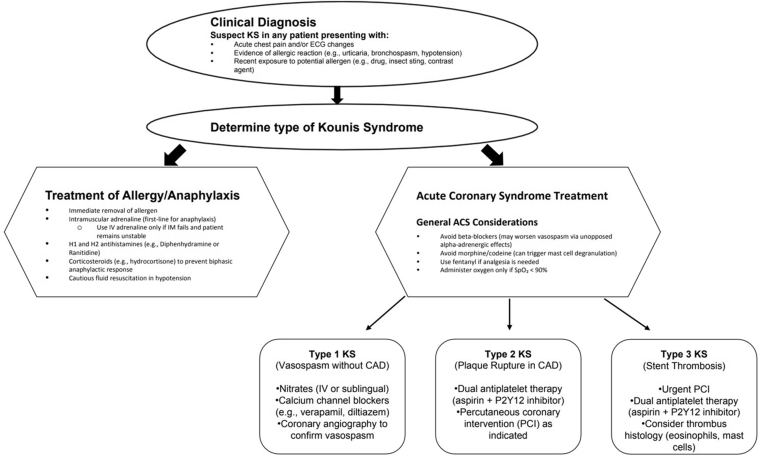
Suggested Treatment Algorithm for Kounis Syndrome ACS = acute coronary syndrome; CAD = coronary artery disease; ECG = electrocardiogram; IV = intravenous; KS = Kounis syndrome; PCI = percutaneous coronary intervention.

## Patient 1

**Figure 2:**
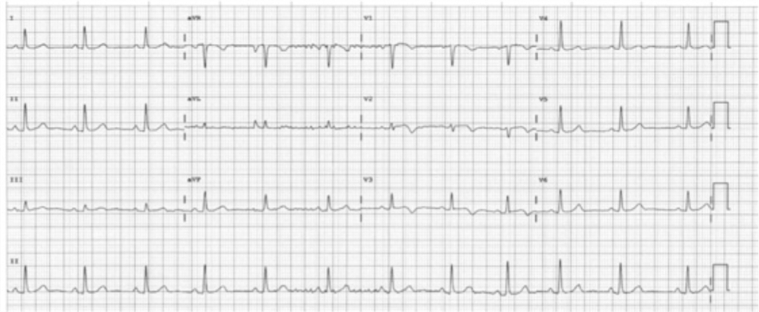
Electrocardiogram of Patient 1 on Presentation to Emergency Department Demonstrating Anterior ST-Segment Elevation (July 24, 2018 at 3:11 pm)

A 52-year-old woman presented to the emergency department with intermittent nonexertional chest pain for 2 weeks. She was of Indian ethnicity, with her only medical history being eosinophilic asthma. Her initial electrocardiogram (ECG) on presentation did not indicate any significant ischemia. As part of the local protocol, she was loaded with aspirin 300 mg in the emergency department. However, this caused a worsening of her chest pains, with subsequent ECGs demonstrating anterior ST-segment elevation (Figure 2).

**Figure 3:**
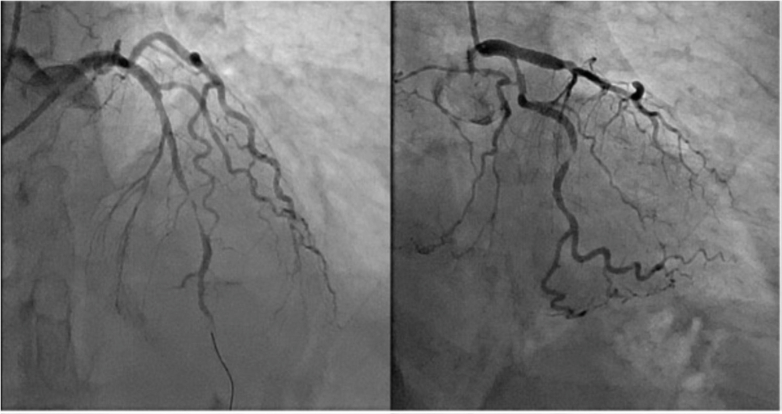
Angiography of Patient 1 Before Intracoronary Glyceryl Trinitrate

**Figure 4:**
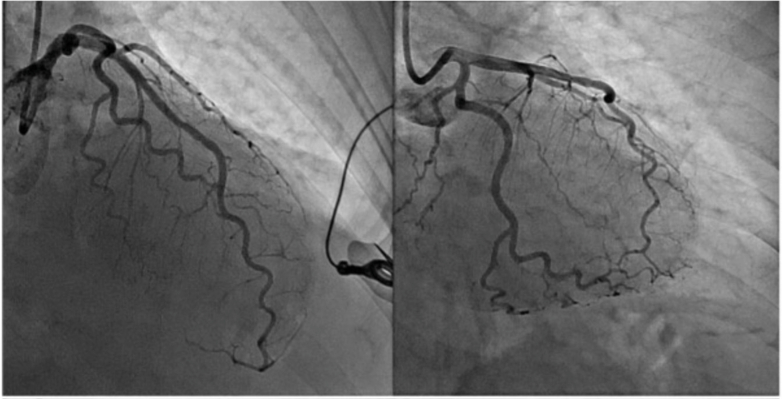
Angiography of Patient 1 After Intracoronary Glyceryl Trinitrate

This led to an emergency left heart catheterization (LHC), showed a severe stenosis of the proximal to mid left anterior descending artery (LAD) and mid left circumflex artery and moderate stenosis of the mid right coronary artery (RCA). However, upon administration of intracoronary glyceryl trinitrate (GTN), these coronary stenoses completely resolved, consistent with a diagnosis of coronary vasospasm precipitated by her aspirin allergy. Hence, no percutaneous coronary intervention (PCI) was required (Figures 3 and 4).

**Figure 5:**
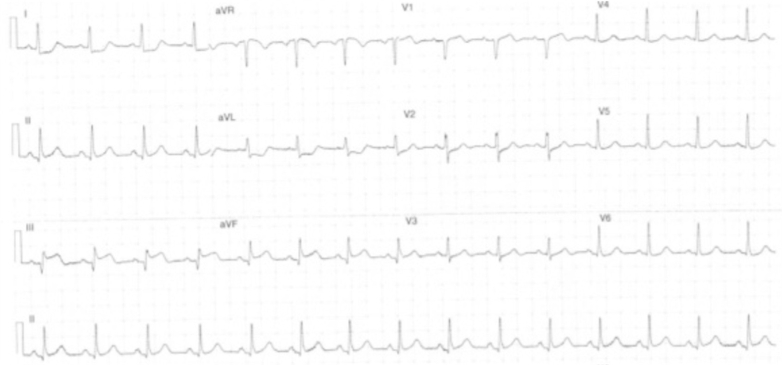
Electrocardiogram of Patient 1 in the Coronary Care Unit on Day 1 After Left Heart Catheterization Sudden-onset chest pain (rated 4 out of 10) resolved with sublingual glyceryl trinitrate. Noting inferior lead ST-segment elevation with reciprocal depression and inferior Q waves.

The patient later reported that she had been given aspirin, which was ceased immediately, with the patient monitored in the coronary care unit during the washout period. During the time, she had occasional chest pains, with dynamic inferior ST-segment elevation that resolved with sublingual GTN (Figure 5).

She was discharged 10 days later. Unfortunately, in this case, tryptase levels were not obtained within 24 hours of admission. However, her eosinophils immediately after ST-segment elevation myocardial infarction were significantly elevated at 1.0 × 10^9^/L. The diagnosis of type 1 KS was made given her history of known aspirin allergy and invasive angiography demonstrating complete reversal of her coronary stenosis with intracoronary GTN. The diagnosis of ACS was made given her troponin I level was significantly elevated at 77.6 ng/L (range, 0-10 ng/L), with cardiac magnetic resonance imaging performed 6 days later showing normal left ventricular ejection fraction with hypokinesis of the basal to mid inferior segments with associated subendocardial and midmyocardial late gadolinium enhancement, in keeping with an RCA-territory infarction. Her autoimmune testing (ANCA, ANA, anti dsDNA, ENA, MPO, PR3) was negative; the opinion of the rheumatology team was sought and agreed with the diagnosis of type 1 KS.

## Patient 2

**Figure 6:**
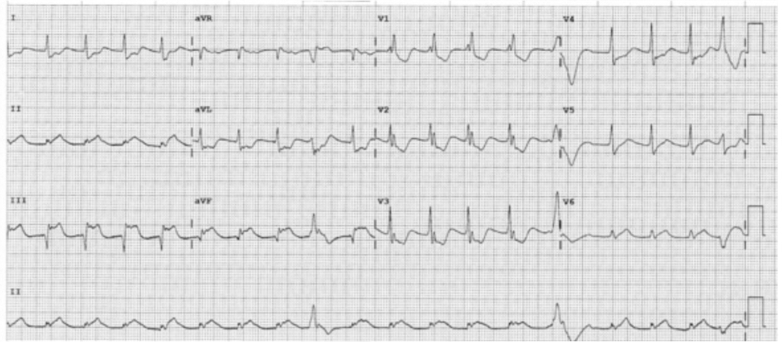
Electrocardiogram of Patient 2 on Arrival to Intensive Care Unit (August 1, 2024 at 2:58 pm)

A 67-year-old man was admitted electively for a laparotomy and adhesiolysis with intraoperative interventional radiology ablation of hepatocellular carcinoma in segment 4a . His medical history was significant for poorly controlled type 2 diabetes, hypertension, dyslipidemia, and metabolic-associated fatty liver disease. Perioperatively, he was administered general anesthesia with intravenous Tazocin (piperacillin-tazobactam) owing to an iatrogenic diaphragmatic breach. During the procedure, there was an episode of profound hypotension and peripheral shutdown that required an adrenaline infusion to manage. This was initially attributed to severe anaphylactic shock secondary to penicillin allergy, which was subsequently confirmed by blood tests demonstrating significantly elevated tryptase levels of 83.9 μg/L (reference: <11.54 μg/L). Upon return to the intensive care unit postoperatively, he remained profoundly hypotensive, requiring significant vasopressor support. ECGs showed inferior ST-segment elevation (Figure 6), and the patiently was urgently transferred to the cardiac catheterization laboratory for LHC.

**Figure 7:**
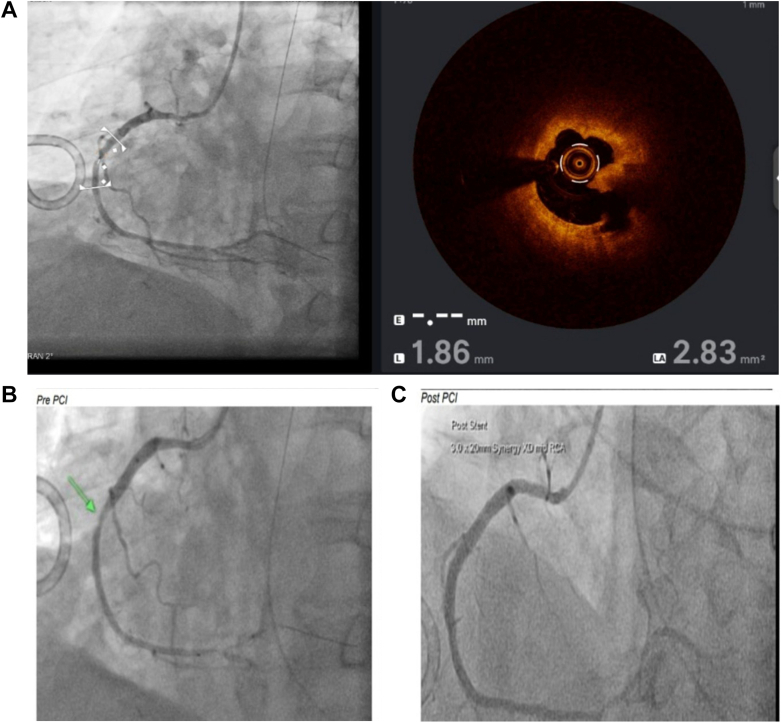
Optical Coherence Tomography and Angiography of Patient 2 (A) Optical coherence tomography demonstrates an eccentric lipid-rich plaque with fibrous cap disruption and cavity formation consistent with plaque rupture. (B and C) Coronary angiography of the right coronary artery on August 1, 2024. Left image depicts pre-PCI status and right image shows post-PCI status. (Drug-eluting stent: 3.0 × 20 mm Synergy XD). PCI = percutaneous coronary intervention.

**Figure 8:**
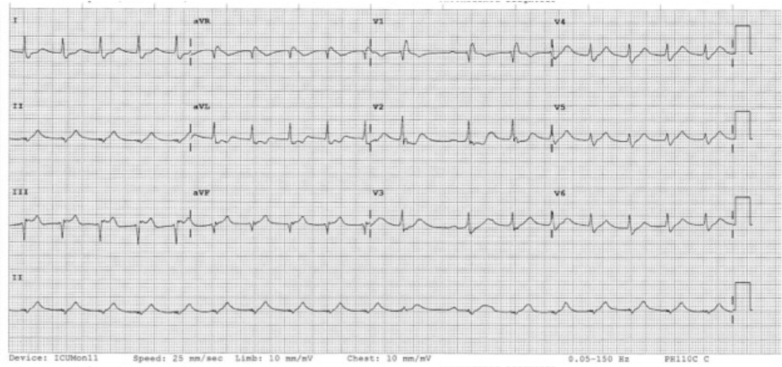
Electrocardiogram of Patient 2 After Left Heart Catheterization With Right Coronary Artery Stent Insertion (August 1, 2024 at 8:22 pm)

This showed a 60% mid-RCA stenosis with plaque rupture. There was also moderate bystander disease in the LAD and left circumflex artery. Optical coherence tomography of the RCA demonstrated evidence of plaque rupture, which was successfully treated with PCI (Figures 7A to 7C). ECG after LHC with RCA stent is shown in Figure 8. Given the significant ECG changes as well as anaphylactic deterioration perioperatively coupled with a raised tryptase level, a diagnosis of type 2 KS was made.

## Patient 3

**Figure 9:**
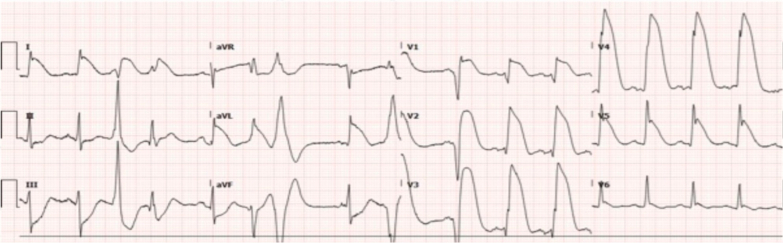
Electrocardiogram of Patient 3 Demonstrating Anterior Myocardial Infarction After Return of Spontaneous Circulation and Defibrillation

Ambulance crews were called to attend to a 59-year-old man in anaphylactic shock. He had a medical history notable for ischemic heart disease with previous coronary stenting. Having experienced multiple simultaneous bee stings, he immediately developed generalized erythema and stridor; his blood pressure was unrecordable on ambulance arrival. He was stabilized on an adrenaline infusion before arrival to the emergency department when he developed chest pain and subsequently entered ventricular tachycardia arrest; return of spontaneous circulation was achieved after a 2-minute downtime with three 200-J synchronized shocks. His ECG indicated anterior myocardial infarction (Figure 9).

**Figure 10:**
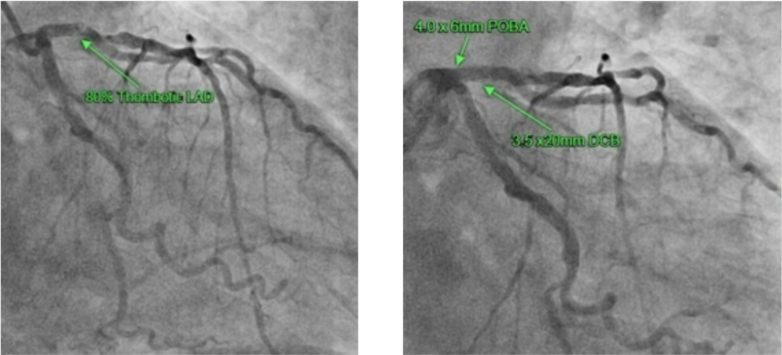
Coronary Angiography of Left Anterior Descending Artery Before and After PCI in Patient 3 Left anterior descending artery (Left) before and (Right) after PCI status displayed. PCI = percutaneous coronary intervention.

He was subsequently loaded with aspirin, heparin, and amiodarone before undergoing an emergent LHC, which revealed 80% in-stent restenosis of his proximal LAD stent; successful PCI of this lesion was performed (Figure 10).

There were no further complications. A tryptase level was taken before LHC and was 128 μg/L (reference: <11.54 μg/L), which normalized within 24 hours in keeping with anaphylaxis. He was discharged with dual antiplatelet therapy and a supply of adrenaline autoinjectors and was referred for ongoing outpatient cardiology and immunology review. This collection of events—anterior myocardial infarction secondary to in-stent restenosis in the setting of anaphylaxis—led to a diagnosis of type 3 KS (Table 1, Table 2, Table 3, Table 4).

## Discussion

Kounis syndrome, a presumptive diagnosis, is based on the coexistence of allergic activation and dynamic coronary pathology. First described in 1991 by Kounis and Zavras, who introduced the term “allergic angina syndrome,”^2^ KS is characterized as an ACS resulting from simultaneous activation of mast cells and platelets in the setting of a hypersensitivity, anaphylactic, or anaphylactoid reaction.^1^^,^^3^^,^^5^ Diagnostic complexity is mirrored in management, which must address both the allergic response and myocardial ischemia. Known triggers include food, medications, environmental exposures, and insect stings.

In the only prospective study of KS thus far, Akoz et al^1^ showed an incidence of 19.4 cases per 100,000 emergency admissions. Medications were the most common precipitant (81%). Despite this, KS remains an under-appreciated cause of ACS among clinicians and is not mentioned as a cause in the latest European Society of Cardiology guidelines.^6^ This likely reflects persistent under-recognition and the lack of large prospective studies supporting formal inclusion. Type 1 KS is the most common variant (72.6%), while type 3 is the least frequent (5.1%).^7^ Mast cell activation is central to the pathophysiology of KS. After IgE-mediated or non–IgE-mediated stimulation, mast cells interact with macrophages, T-lymphocytes, and platelets through coordinated inflammatory signaling pathways. Degranulation results in the release of mediators including histamine, tryptase, cytokines, and arachidonic acid–derived products such as leukotrienes, platelet-activating factor, thromboxane, prostacyclin, and tumor necrosis factor–α. These mediators contribute to coronary vasomotor dysfunction, plaque destabilization, and coronary thrombogenesis. The relevance of these mechanisms is supported by the high density of mast cells within both myocardial tissue and the coronary arterial wall, particularly in regions adjacent to atherosclerotic plaques.^3^ Furthermore, elevated serum tryptase has been observed during spontaneous myocardial ischemia but not during pharmacologically induced coronary spasm, supporting a mechanistic role for mast cell activation in KS.^4^

Clinically, KS presents with hypersensitivity or anaphylaxis alongside acute myocardial ischemia, which may persist after shock resolution. Myocardial ischemia is defined by ischemic symptoms, new ECG changes, imaging evidence of loss of viable myocardium or regional wall motion abnormalities, or coronary thrombus. It may occur without necrosis or progress to myocardial infarction, defined by a rise/fall in cardiac troponin above the 99th percentile.^8^

In type 1 KS, coronary vasospasm results in an imbalance between myocardial oxygen supply and demand, consistent with type 2 myocardial infarction when myocardial necrosis occurs. In contrast, type 2 and type 3 KS are associated with plaque rupture or stent thrombosis, resulting in type 1 myocardial infarction. These mechanisms were illustrated in our 3 cases.

In patient 1, inadvertent administration of aspirin in the setting of aspirin hypersensitivity precipitated anterior ST-segment elevation with angiographic evidence of LAD stenosis. KS occurs via proposed mechanisms distinct from systemic anaphylaxis; unlike classic anaphylaxis, it preferentially affects the coronaries rather than causing systemic vasodilation. Histamine plays a critical role by inducing coronary vasoconstriction, upregulating tissue factor expression, and activating platelets, thereby promoting vasospasm and thrombosis. Supporting this, circulating histamine concentrations in hypersensitivity-associated ACS have been shown to be more than twice those observed in matched patients with ACS of nonallergic etiology.^2^^,^^3^^,^^9^ In aspirin-sensitive individuals, exposure may trigger mast cell activation through non–IgE-mediated mechanisms, resulting in disproportionate local mediator release within the coronary circulation. In the present case, vasospasm resolved with intracoronary GTN; however, the patient sustained a type 2 myocardial infarction, demonstrating that type 1 KS is not a benign entity despite its reversibility.

In patients 2 and 3, anaphylactic reactions to penicillin derivatives and bee stings, respectively, occurred in the setting of pre-existing coronary artery disease. In both cases, allergic activation precipitated plaque rupture and stent thrombosis, confirmed by optical coherence tomography, resulting in type 1 myocardial infarction requiring PCI.

Diagnostic certainty was limited in patient 1 by the absence of serum tryptase measurement within the optimal 24-hour window. While peripheral eosinophilia supported an allergic response, it is not the biochemical gold standard for mast cell activation. In contrast, diagnoses in patients 2 and 3 were supported by elevated tryptase levels, strengthening the attribution of mast cell–mediated hypersensitivity. This variability reflects real-world practice and the challenges of obtaining timely biochemical confirmation of KS.

A key learning point from this case series is that management of KS differs from conventional atherosclerotic ACS. Currently, there are no dedicated treatment guidelines for KS, and the supporting evidence is low quality, consisting largely of case reports and series. Nonetheless, management should aim to restore myocardial perfusion while addressing the concurrent allergic reaction, with treatment tailored to the KS subtype. A pragmatic management algorithm is therefore proposed (Figure 1), intended to support clinical decision-making rather than serve as a guideline-level recommendation.

In type 1 KS, treatment targets the allergic reaction and coronary vasospasm reversal, with prompt identification and removal of triggers. Supportive therapy includes the use of H1 and H2 antihistamines, such as diphenhydramine (1-2 mg/kg) or ranitidine (1 mg/kg) in the immediate setting to decrease further allergic manifestations. In addition, the use of corticosteroids, for example hydrocortisone 1 to 2 mg/kg/d, may be beneficial in preventing a biphasic anaphylactic response. Although corticosteroids can impair wound healing and theoretically increase the risk of myocardial thinning, aneurysm, or rupture, their use in ACS have not been associated with increased mortality or harm.^4^

In anaphylaxis-induced distributive shock, fluid resuscitation is essential but must be balanced to avoid pulmonary edema in ACS. Furthermore, adrenaline is lifesaving first-line therapy in anaphylaxis but may exacerbate myocardial ischemia, prolong QTc, and precipitate arrhythmias or vasospasm, particularly with intravenous administration.^10^ Therefore, in the first instance, adrenaline should be administered intramuscularly in a dose of 0.2 to 0.5 mg (1:1,000) every 5 minutes until symptom resolution. Intravenous adrenaline should be reserved for patients unresponsive to repeated intramuscular treatment or persistent hemodynamic or respiratory instability.

In contrast to traditional management of ACS, beta-blockers are contraindicated in ACS secondary to KS, as they may precipitate unopposed alpha-adrenergic effect, worsening myocardial ischemia and vasospasm.^6^ In patients taking beta-blockers with worsening ischemia, glucagon may be considered to counteract beta-blocker effects.^4^^,^^7^

Similarly, opioids (codeine, morphine, meperidine) may increase mast cell degranulation and should be used cautiously in KS.^4^ Fentanyl and its derivatives are less likely to induce mast cell activation and should be preferred.^3^^,^^9^ In the acute phase, particularly in types 1 and 2, the mainstay of coronary vasospasm therapy is the use of nitrates (either oral or intravenous) to relieve coronary spasm and restore myocardial oxygenation. Calcium-channel blockers, such as verapamil or diltiazem, are suitable adjuncts to relieve vasospasm. Coronary angiography is recommended to confirm vasospasm and exclude plaque rupture.

If PCI is required (ie, in type 2 or 3 KS), dual antiplatelet therapy comprised of acetylsalicylic acid and a second P2Y_12_ receptor inhibitor should be administered. Oxygen supplementation is only recommended in the setting of hypoxia (oxygen saturation <90%).^6^

In summary, Kounis syndrome remains a rare but clinically significant cause of ACS that presents in several forms. Early recognition and differentiation from other ACS causes are critical, as standard ACS therapy may exacerbate cardiac injury. Treatment should be tailored to KS type, addressing both the allergic reaction and myocardial perfusion, with multidisciplinary input from cardiology and allergy/immunology.

## Funding Support and Author Disclosures

The authors have reported that they have no relationships relevant to the contents of this paper to disclose.
